# Does the total consumption model apply to cannabis use?

**DOI:** 10.1111/add.70353

**Published:** 2026-02-24

**Authors:** Thor Norström, Håkan Leifman

**Affiliations:** ^1^ Swedish Institute for Social Research Stockholm University Stockholm Sweden; ^2^ Department of Clinical Neuroscience Karolinska Institutet Stockholm Sweden

**Keywords:** adolescents, cannabis, distribution, Sweden, theory of collectivity, total consumption mode

## Abstract

**Aims:**

To test whether the total consumption model and its extension, the theory of collectivity, apply to adolescent cannabis use. We estimated: (1) whether the distribution of cannabis use frequency was stable over time; (2) whether changes in average cannabis use were mirrored across consumption quantiles; and (3) whether higher average use corresponds with a higher prevalence of high‐frequency users.

**Design:**

Repeated cross‐sectional study based on annual surveys. We analyzed trends in cannabis use frequency across consumption percentiles using Lorenz curves and Gini coefficients to assess distributional stability. We used ordinary least squares (OLS) regression to estimate the elasticities between overall mean frequency of use and mean frequency of use for five quantiles ranging from P25 to P95. The association between average use and the prevalence of high‐frequency users was examined graphically and parametrically.

**Setting:**

Sweden.

**Participants:**

Ninth‐grade students (aged 15–16) surveyed annually between 1990 and 2023 (excluding 2013), with a total of 180 059 respondents, and 2nd‐year high school students (aged 17–18) surveyed annually between 2004 and 2023 (excluding 2013 and 2020), totaling 80 925 respondents. Our analyses were limited to individuals who reported cannabis use, resulting in analytical samples of 10 139 9th‐grade students and 11 160 2nd‐year high school students.

**Measurement:**

Frequency of cannabis use was measured by a question on how many occasions the respondent has used hashish or marijuana. The seven response alternatives ranged from 0 to 50 times or more.

**Findings:**

Mean frequency of cannabis use fluctuated statistically significantly across survey years among 9th‐grade students (F‐test = 7.647, *P* < 0.001) and marginally among 2nd‐year students (F‐test = 1.550, *P* = 0.068). Notwithstanding this variation in mean use, the distribution of frequency of cannabis use remained stable: Lorenz curves were consistent across years, and Gini coefficients showed no significant changes. Mean frequency in all five quantiles (25th–95th) were positively and statistically significantly associated with overall mean frequency, suggesting synchronized changes across user groups; e.g. the elasticity for P50 was estimated at 0.914 (*P* < 0.001) in 9th grade. Increases in mean frequency use were associated with a higher prevalence of high‐frequency users. Thus, an increase in average frequency of cannabis use by 1 percentage point was associated with a 1.794 (standard error = 0.065, *P* < 0.001) percentage point increase in high‐frequency users in 9th grade.

**Conclusions:**

Adolescent cannabis use in Sweden appears to conform to key predictions of the total consumption model and its extension, the theory of collectivity.

## INTRODUCTION

Over the past two decades, many countries have liberalized their cannabis policies, through measures such as decriminalization, medical access and full legalization. These policy shifts have often been followed by increases in cannabis use, particularly in jurisdictions where legal access has expanded [1, 2, 3, 4]. Yet, the underlying dynamics of these increases remain insufficiently understood. A key unresolved question is whether rising cannabis use reflects a general upward shift across the whole population of users or whether it is driven primarily by growth within specific subgroups—such as high‐frequency users. To address this issue, we analyze repeated cross‐sectional survey data on adolescent cannabis use in Sweden, a context where cannabis remains illegal but where public discourse and attitudes toward the drug have become more permissive over time.

A useful theoretical point of departure is the groundbreaking work of Rose and Day [5], who demonstrated a close link between the population mean of health‐related behaviors and the prevalence of associated problems. Their core argument is that ‘distributions of health‐related characteristics move up and down as a whole’, implying that changes in problem prevalence cannot be understood in isolation from changes in population‐level behavior. This reasoning has been particularly influential in alcohol research, where it forms the basis of what came to be known as the total consumption model (TCM) [6]. An early articulation of the model appeared in a classic WHO report from 1975 [7], which highlighted the strong association between per capita alcohol consumption and liver cirrhosis mortality, thereby underscoring the importance of total consumption in society. The key message of the TCM is that prevention efforts cannot be confined to high‐risk groups but must also address the population at large.

However, while the TCM establishes a link between mean consumption and excessive drinking in society, it does not lay out the mechanisms behind this relationship. The Norwegian sociologist Ole‐Jørgen Skog elaborated the TCM by providing a theoretical foundation in his theory of the collectivity of drinking behavior [8]. Drawing on extensive alcohol survey data, Skog demonstrated that changes in per capita consumption tend to be collective in nature: shifts in the average are reflected throughout the distribution of drinkers, from light to heavy users. He pointed to two main mechanisms that can account for this pattern: (i) social network effects, whereby individuals adjust their consumption in response to that of their peers; and (ii) common exogenous influences, such as changes in policy, availability or pricing.

Skog's model of collectivity has received empirical support in multiple studies of both adult [9, 10] and youth [11, 12] alcohol use. However, not all research aligns with this collective shift hypothesis. Some studies point to a polarization of consumption, in which changes are concentrated in specific segments of the population. For example, while the overall trend in youth drinking has been downward in many countries, evidence from Sweden suggests that a small group of heavy‐drinking adolescents may be increasing their consumption during this general decline [13]. A parallel has been observed in research on body mass index (BMI), where the rising average BMI is accompanied by particularly steep increases in the upper tail of the distribution [14], challenging the generalizability of Rose's hypothesis [15].

As noted above, a central assumption of Skog's model of collectivity is that individual behavior is shaped by social influences. In the present context, this implies that an adolescent's cannabis use is affected by the cannabis use of their peers. This assumption is supported by a substantial body of research. Thus, a systematic review of longitudinal studies has confirmed that peer cannabis use is a strong predictor of individual use [16], while also controlling for homophily and other confounding factors. Moreover, evidence from a related domain—tobacco smoking—suggests that such network effects can influence population‐level outcomes [17].

Alongside peer influence, shifting cultural norms have likely contributed to changes in cannabis behavior. Cannabis has become increasingly socially acceptable, and perceptions of its harmfulness have declined among both adolescents and adults [18, 19]. These attitudinal changes may facilitate more widespread and frequent use, particularly among youths. This is of particular concern from a public health standpoint, as regular cannabis use during adolescence—when brain development is ongoing—has been associated with a range of negative outcomes, including cognitive deficits and increased risk for psychiatric disorders [20].

### Research questions

If the TCM, and its extension in the theory of collectivity, apply to adolescent cannabis use, several testable implications follow.

1. The distribution of cannabis use frequency should remain stable over time, even as the average frequency of use shifts. More specifically, we expect the distribution to retain a similar shape and relative dispersion, as indicated by Lorenz curves and the Gini coefficient, across years.
2. There should be a positive association between the overall average frequency and average frequency of use within each subgroup, from infrequent to very frequent users.
3. The average frequency of cannabis use should be positively associated with the prevalence of high‐frequency users.

The aim of this study is to empirically test these implications using repeated cross‐sectional data on cannabis use among Swedish adolescents.

We conducted a literature search to identify studies similar to ours. First, we ran the following search string in Google Scholar: (‘total consumption model’ OR collectivity) AND (cannabis OR marijuana OR hashish). None of the results were relevant. Second, we reviewed all studies citing Skog (1985) [8] (*n* = 686). Three of these [21, 22, 23] addressed cannabis or marijuana, but none engaged with the specific research questions examined in the present study.

## METHODS

### Data

This study draws on data from the annual school surveys conducted by the Swedish Council for Information on Alcohol and Other Drugs (CAN). These surveys investigate substance use and related issues among Swedish school students and represent the longest‐running annual school survey on substance use in Europe. Since 1971, CAN has surveyed 9th‐grade students (aged 15–16 years), and since 2004 it has also included 2nd‐year high school students (equivalent to 11th grade, aged 17–18 years).

For this study, we use data from 1990 to 2023 for 9th‐grade students, which is the period for which digitalized data are available, and from 2004 to 2023 for 2nd‐year high school students. However, we discarded the data for 2013, because the response alternatives for the cannabis question were framed differently that year. Further, the 2020 wave is missing for the 2nd‐year high school students owing to COVID‐19‐related school closures.

From 1990 to 2018, the survey was administered as an anonymous paper‐and‐pencil questionnaire, completed in the classroom under teacher supervision during March or April each year. In 2019, schools could choose between paper or a web form, and since 2021 the survey has been conducted entirely in digital format, but still based in the classroom. An evaluation of the 2019 survey found no systematic differences in prevalence estimates between paper and web administration, in either 9th grade or 2nd‐year high school [24].

Each annual wave includes about 5000 9th‐grade and 4300 2nd‐year high school students (with a roughly equal gender distribution). A two‐stage stratified sampling procedure ensured national representativeness: in the first stage, schools were selected with probability proportional to size (PPS), and in the second stage, one class was randomly chosen within each selected school. The median response rate among sampled classes was 84% (range = 69%–96%) in 9th grade and 79% (range = 70–87%) in the 2nd year of high school. At the individual level (all students enrolled in the selected classes), the median response rate was 85% (range = 78%–90%) in 9th grade and 82% (range = 79–86%) in the 2nd year of high school. Missing responses on cannabis frequency were rare (≈1.0% and ≈0.9%, respectively).

The total number of respondents was 180 059 for 9th‐grade and 80 925 for 2nd‐year high school students. Because our analyses include only respondents who have used cannabis, our analytical sample is markedly attenuated. For 9th‐grade students it amounts to 10 139 individuals in total, while the corresponding number for 2nd‐year high school students is 11 160 (for an annual breakdown, see Tables 1 and 2). The data have been cleaned and harmonized to ensure consistency across years.

**TABLE 1:** Descriptive statistics for frequency of cannabis use in 9th grade of junior high school.

Year	*n*	% users	Frequency of cannabis use			Median	IQR	Gini	
			Mean	SE	SD			EST	SE
1990	196	3.305	8.324	1.039	14.545	3	6.5	0.694	0.046
1991	183	3.127	6.615	0.913	12.354	3	6.5	0.677	0.055
1992	211	3.625	7.002	0.854	12.406	3	6.5	0.675	0.047
1993	246	4.190	7.065	0.826	12.961	3	2.0	0.682	0.046
1994	247	4.248	5.696	0.657	10.324	3	6.5	0.646	0.047
1995	298	5.381	5.601	0.605	10.437	3	2.0	0.636	0.045
1996	405	6.812	6.091	0.527	10.614	3	2.0	0.651	0.034
1997	402	7.126	7.106	0.617	12.362	3	6.5	0.659	0.034
1998	376	6.981	7.684	0.691	13.408	3	6.5	0.664	0.036
1999	402	7.809	6.591	0.598	11.994	3	6.5	0.663	0.037
2000	391	7.426	8.005	0.702	13.872	3	6.5	0.676	0.034
2001	431	7.955	8.586	0.669	13.881	3	6.5	0.659	0.029
2002	383	7.083	9.051	0.780	15.271	3	6.5	0.672	0.033
2003	324	6.211	9.568	0.861	15.494	3	6.5	0.676	0.033
2004	347	6.458	9.893	0.867	16.154	3	6.5	0.681	0.032
2005	313	5.821	8.698	0.833	14.737	3	6.5	0.681	0.036
2006	238	4.884	8.828	0.980	15.112	3	6.5	0.681	0.042
2007	246	4.679	9.203	0.946	14.841	3	6.5	0.668	0.038
2008	259	5.352	8.981	0.954	15.346	3	6.5	0.682	0.040
2009	355	6.869	10.363	0.886	16.702	3	6.5	0.681	0.031
2010	352	7.329	9.464	0.816	15.308	3	6.5	0.666	0.032
2011	320	6.966	11.503	0.924	16.521	3	14.5	0.649	0.027
2012	272	6.053	12.250	0.634	18.105	3	14.5	0.601	0.026
2014	324	6.679	12.718	1.018	18.316	3	14.5	0.655	0.026
2015	273	5.592	12.350	1.061	17.523	3	12.5	0.637	0.029
2016	236	4.987	15.108	1.276	19.604	3	14.5	0.629	0.024
2017	341	5.651	13.587	1.008	18.611	3	14.5	0.640	0.023
2018	322	6.158	12.870	1.002	17.976	3	14.5	0.648	0.025
2019	319	6.175	11.440	0.966	17.252	3	6.5	0.659	0.029
2020	245	6.017	12.851	1.166	18.254	3	14.5	0.653	0.029
2021	269	5.175	10.152	0.974	15.969	3	6.5	0.667	0.034
2022	288	5.555	13.743	1.110	18.833	3	13.5	0.642	0.025
2023	303	5.902	11.682	0.997	17.357	3	6.5	0.662	0.029
*F*‐test			7.647					0.011	
*P*			<0.001					>0.999	

**TABLE 2:** Descriptive statistics for frequency of cannabis use in 2nd grade of high school.

Year	*n*	% users	Frequency of cannabis use			Median	IQR	Gini	
			Mean	SE	SD			EST	SE
2004	597	13.362	10.446	0.637	15.570	3	6.5	0.652	0.021
2005	630	13.889	11.106	0.674	16.912	3	6.5	0.667	0.021
2006	528	14.103	11.362	0.733	16.835	3	14.5	0.660	0.022
2007	616	14.382	10.343	0.634	15.738	3	6.5	0.653	0.022
2008	571	14.430	9.334	0.617	14.753	3	6.5	0.663	0.024
2009	635	15.718	10.995	0.668	16.825	3	6.5	0.664	0.021
2010	675	17.312	12.428	0.680	17.656	3	14.5	0.646	0.018
2011	576	16.157	12.138	0.707	16.978	3	14.5	0.639	0.019
2012	547	16.471	12.152	0.743	17.388	3	14.5	0.606	0.016
2014	602	15.880	13.323	0.757	18.569	3	14.5	0.587	0.013
2015	586	14.210	13.078	0.755	18.276	3	14.5	0.645	0.018
2016	660	16.633	12.540	0.696	17.874	3	14.5	0.643	0.019
2017	734	15.604	11.689	0.633	17.152	3	6.5	0.649	0.018
2018	697	14.503	12.049	0.649	17.142	3	14.5	0.653	0.018
2019	683	14.777	12.463	0.678	17.714	3	14.5	0.641	0.018
2021	556	12.888	11.911	0.754	17.767	3	14.5	0.645	0.018
2022	624	12.981	12.697	0.727	18.149	3	14.5	0.661	0.022
2023	643	12.713	11.159	0.657	16.657	3	6.5	0.655	0.018
*F*‐test			1.550					0.021	
*P*			0.068					>0.999	

### Measures

Respondents who reported any drug use in the initial screening question were asked a follow‐up item on their frequency of cannabis use. Since 2012, the screening options have been ‘No’, ‘Yes, during the past 30 days’, ‘Yes, during the past 12 months’ and ‘Yes, more than 12 months ago’; prior to 2012, the options were ‘Yes/No’. In both cases, all respondents answering ‘Yes’ were subsequently presented with the question on frequency of cannabis use.

Cannabis use was thus measured as lifetime use. Frequency of cannabis use was measured by the question: ‘On how many occasions have you used hashish or marijuana?’. The response alternatives were: 0 (coded 0), 1 (coded 1), 2–4 (coded 3), 5–10 (coded 7.5), 11–20 (coded 15.5), 21–50 (coded 35.5) and 50 times or more (coded 55).

Although lifetime use is not identical to recent use, it provides a reasonable proxy in adolescent populations. Published data from CAN [25] show that the discrepancy between lifetime and past‐year prevalence of any illicit drug use (though not specifically cannabis) is small, particularly in the 9th grade, and that initiation before the age of 14 years is rare. Lifetime use can therefore be regarded as a feasible indicator of recent cannabis use when analyzing distributional patterns.

### Statistical analyses

**FIGURE 1:**
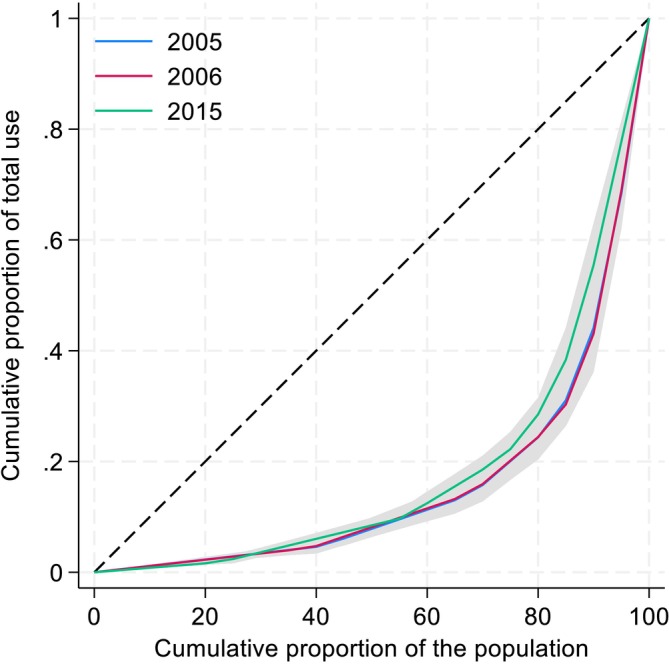
Lorenz curves with 95% confidence intervals for frequency of cannabis use for the years 2005, 2006 and 2015 in 9th‐grade students.

**FIGURE 2:**
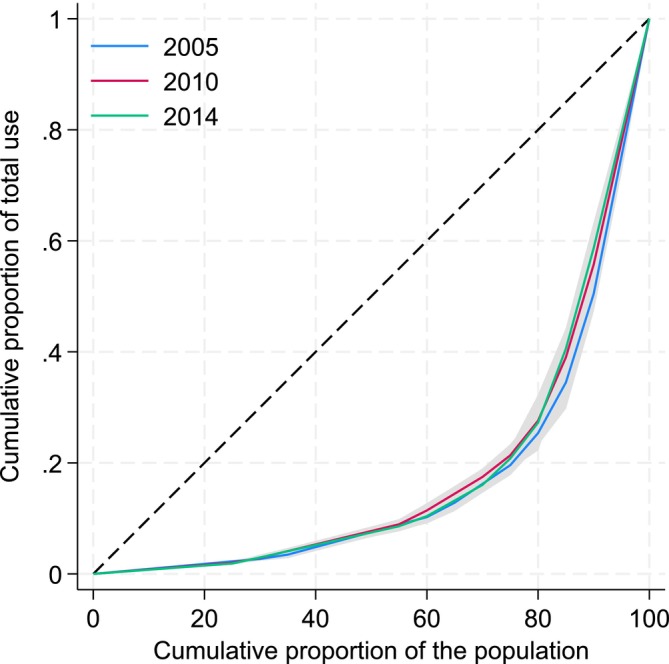
Lorenz curves with 95% confidence intervals for frequency of cannabis use for the years 2005, 2010 and 2014 in 2nd‐year high school students.

To assess the stability of the distribution of frequency of cannabis consumption over time, we began by visually inspecting Lorenz curves. A Lorenz curve graphically represents the distribution of a variable—in this case, frequency of cannabis consumption—by plotting cumulative population percentiles (ranked by frequency level) on the horizontal axis against the corresponding cumulative share of total frequency on the vertical axis [26]. A 45° reference line represents a distribution with perfect equality. A stable shape of the curves over time would suggest a time‐invariant distribution for frequency of use, whereas changes in the shape would indicate changes in the relative dispersion. The Lorenz curves were estimated by the command *lorenz* in Stata, which also provides estimated standard errors and 95% confidence intervals to enable the assessment of whether the distribution is stable over time. We present Lorenz curves for 3 years (Figures 1 and 2): the year with the lowest Gini coefficient, the year with the highest Gini coefficient and an intermediate year (Lorenz curves for all years are displayed in Figures S1 and S2). To supplement this graphical analysis, we computed Gini coefficients for each year. The Gini coefficient is defined as the ratio of the area between the Lorenz curve and the 45° line to the total area under the 45° line. The coefficient thus provides a summary measure of inequality (or skewness) in the distribution [26], with higher values indicating a more unequal distribution of frequency of cannabis use (i.e. use concentrated among fewer individuals). Further, the Gini coefficient is scale‐invariant—proportional changes in all values (and hence the mean) leave it unchanged—so year‐to‐year differences in the coefficient reflect changes in relative dispersion/shape rather than level. Using the standard errors of the Gini coefficients we computed *F*‐tests to assess the degree of homogeneity in Gini coefficients across time.

To test the hypothesis of collectivity in cannabis consumption—that is, whether consumption levels change in tandem across user groups—we estimated the frequency of cannabis consumption of the 25th, 50th, 75th, 90th and 95th percentiles based on the cannabis frequency distributions extracted from the annual survey data. These percentiles indicate the frequency level of the whole spectrum of consumption groups, from low‐ to high‐frequency consumers.

**FIGURE 3:**
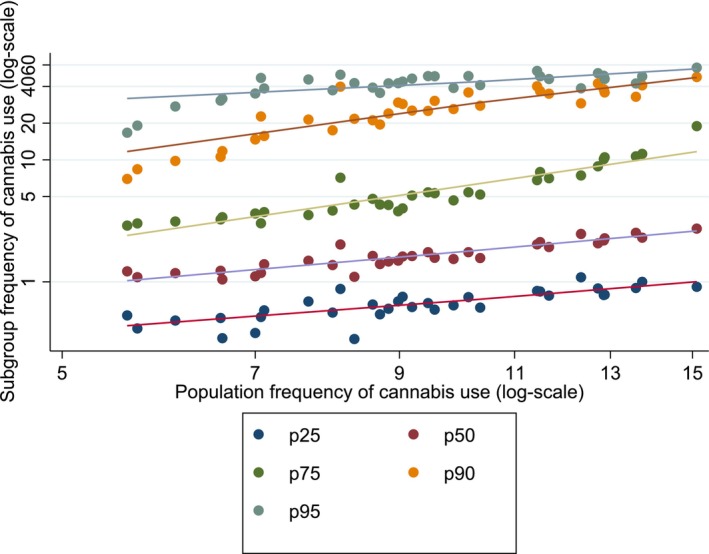
Frequency of cannabis use in five consumption groups (defined by percentiles) plotted against overall mean frequency. Based on annual data 1990–2023 (excluding 2013) for 9th‐grade students. The straight lines are fitted with ordinary least square regression.

**FIGURE 4:**
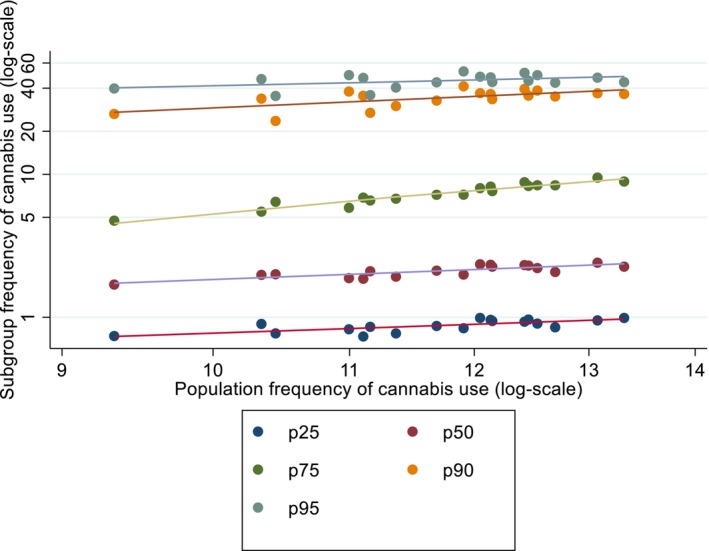
Frequency of cannabis use in five consumption groups (defined by percentiles) plotted against overall mean frequency. Based on annual data 2004–2023 (excluding 2013 and 2020) for 2nd‐year high school students. The straight lines are fitted with ordinary least square regression.

Following Skog's [8] approach to testing collectivity in alcohol consumption, we used ordinary least squares (OLS) regression to estimate the following model for each of the five consumption groups:

(1) $$\mathrm{Ln}C_{\mathrm{it}}=a_{i}+e_{i}*{\mathrm{LnC}}_{t},$$

where *C*_*it*_ is the mean consumption in consumption group *i* at year *t*, and *C*_*t*_ is the overall mean consumption at year *t*. *Ln* signifies the natural logarithm. The parameter of interest is *e*_*i*_; that is, the elasticity that expresses the percentage change in mean consumption in consumption group *i*, given a 1% change in overall mean consumption. The collectivity hypothesis predicts that that the elasticity is positive for each of the consumption groups, reflecting synchronized shifts across the consumption distribution. The estimated regression lines are displayed in Figures 3 and 4.

The relationship between the average frequency of cannabis use and the prevalence of high‐frequency cannabis use (defined as ≥20 times) was assessed graphically, and through regression analysis.

Only cannabis users were included in the analyses. All statistical analyses were performed with Stata 18 (StataCorp LLC, College Station, TX, USA). The study was not pre‐registered, thus the results should be considered exploratory.

## RESULTS

The results are presented in three parts, corresponding to the main research questions: (1) the stability of the frequency of cannabis consumption distribution over time; (ii) the synchronization of consumption across user groups; and (iii) the relationship between overall use and the prevalence of high‐frequency use.

### Stability of the frequency of cannabis consumption distribution

Figures 1 and 2 depict the Lorenz curves for the 9th‐grade and 2nd‐year high school students, respectively. These curves suggest that the distribution of the frequency of cannabis consumption has remained quite stable over time in both age groups. This impression is supported by the estimated Gini coefficients shown in Tables 1 and 2. The Gini values remain relatively constant throughout the study period, and the *F*‐tests do not indicate significant shifts in the overall distribution. This suggests that the inequality in the frequency of consumption remained unaltered during the study period, despite significant changes in average cannabis use among 9th‐grade students (*F*‐test = 7.647, *P* < 0.001) and near‐significant changes in average cannabis use among 2nd‐year high school students (*F*‐test = 1.550, *P* = 0.068).

### Synchronization of use across the consumption spectrum

The next set of analyses examines whether changes in mean cannabis use are mirrored across different consumption levels—a core prediction of the theory of collectivity.

Figures 3 and 4 suggest that all consumption groups tend to move in tandem with changes in the population average. In other words, as average cannabis use increases or decreases, similar changes are observed across low, moderate and heavy use percentiles—supporting the idea of collectivity in adolescent cannabis consumption. This interpretation is reinforced by the regression results in Table 3, where the elasticity estimates for each consumption group are presented. The coefficients are positive and statistically significant for all groups. For instance, the elasticity for the fiftieth percentile (P50) was estimated at 0.914 (*P* < 0.001) in 9th grade.

**TABLE 3:** Relationship between overall mean frequency of consumption and mean frequency of consumption in various consumption groups (percentiles) estimated as elasticity (*e*) (with robust SE).

Percentile	9th grade junior high school			2nd‐year high school students		
	*e*	SE	*P*	*e*	SE	*P*
25	0.864	0.104	<0.001	0.784	0.151	<0.001
50	0.914	0.064	<0.001	0.887	0.118	<0.001
75	1.594	0.137	<0.001	1.950	0.129	<0.001
90	1.655	0.159	<0.001	1.087	0.281	0.001
95	0.731	0.166	<0.001	0.555	0.232	0.030

Supplementary exploratory analyses indicated that the correlations between mean frequency of consumption among users and the proportion of users were weak and statistically non‐significant (9th grade, *r* = 0.111, *P* = 0.540; 2nd‐year high school students, *r* = 0.323, *P* = 0.191). Thus, our data provide no evidence of a relationship between these two measures.

### Relationship between overall use and the prevalence of high‐frequency users

**FIGURE 5:**
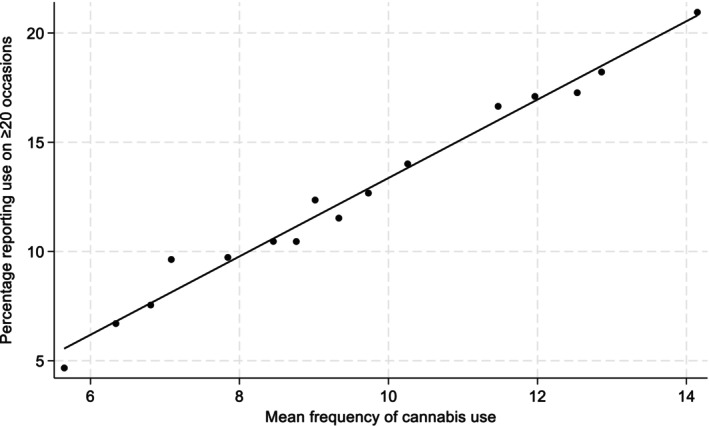
Percentage of high‐frequency cannabis consumers (≥20 times) plotted against mean frequency of cannabis use. Based on annual data 1990–2023 (excluding 2013) for 9th‐grade students (annual values averaged over 2 years).

**FIGURE 6:**
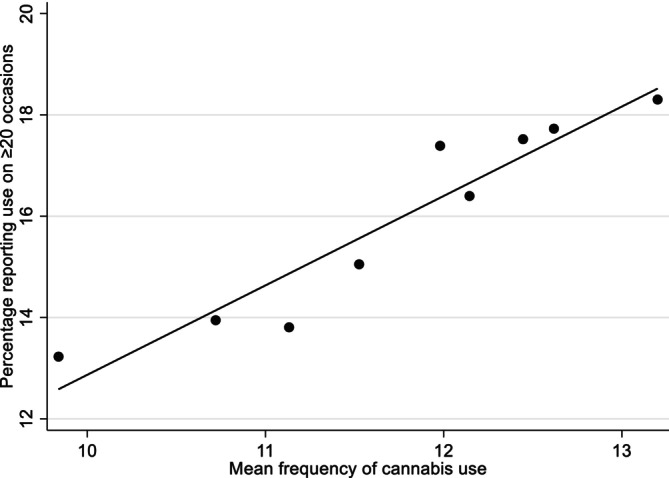
Percentage of high‐frequency cannabis consumers (≥20 times) plotted against mean frequency of cannabis use. Based on annual data 2004–2023 (excluding 2013 and 2020) for 2nd‐year high school students (annual values averaged over 2 years).

Figures 5 and 6 show a marked and positive association between overall frequency of cannabis use and the prevalence of high‐frequency users, indicating that periods of elevated average use coincide with an increased share of users reporting very frequent cannabis consumption. Ordinary least‐square regressions suggested that an increase in average frequency of cannabis use by one percentage point was associated with a 1.794 (SE = 0.065, *P* < 0.001) percentage point increase in the prevalence of high‐frequency users among 9th‐grade students. The corresponding number for 2nd‐year high school students was 1.765 (SE = 0.228, *P* < 0.001).

Taken together, these findings lend strong support to the TCM and its extension, the theory of collectivity, in the context of adolescent cannabis use. The results suggest that population‐level changes in cannabis use are not confined to specific user groups but are instead broadly reflected across the distribution. The relative stability of the consumption distribution and the observed synchronization of trends point to the importance of population‐level dynamics—such as peer influence, changing norms and policy environments—in shaping adolescent cannabis use patterns.

## DISCUSSION

### Summary and interpretation of findings

The TCM was originally developed in the context of alcohol and public health, but has since been applied to both gambling [27] and medicinal drug use [28]. Although previous studies have documented the highly skewed distribution of cannabis consumption among users [22, 29, 30], this study is, to our knowledge, the first to examine whether the TCM and its extension, the theory of collectivity of change, can be applied to cannabis use. Our findings suggest that, broadly speaking, the core assumptions of these frameworks hold for adolescent cannabis use in Sweden. In particular, we find that: (i) the distribution of frequency of cannabis use remains stable over time; (ii) the various consumption groups display a synchronized response to changes in overall mean use; and (iii) higher levels of frequency of consumption are associated with a higher prevalence of high‐frequency users.

The stability of the cannabis frequency distribution across time, as shown by the Lorenz curves and Gini coefficients, indicates that increases or decreases in mean cannabis use do not substantially alter the shape of the distribution. This is a key prediction of the TCM, which posits that changes in average use reflect near proportional shifts across the entire user spectrum, rather than being driven solely by specific subgroups such as heavy users. The results suggest that while the overall level of cannabis use may vary from year to year, the relative proportions of use across the population remain quite consistent.

Our second set of analyses supports the notion of *collectivity* in cannabis use. Consistent with Skog's exposition of the TCM [8], we find that all consumption groups—ranging from low to heavy users—adjust their consumption in tandem with changes in the population mean.

The third finding—that periods of increased frequency of use are associated with a higher prevalence of high‐frequency users—further aligns with the predictions of the TCM. This relationship underscores a key public health implication: even modest increases in overall cannabis use at the population level can translate into a larger number of individuals engaging in high‐risk patterns of consumption. In other words, population‐level trends are meaningful indicators of cannabis‐related harm.

Taken together, these findings suggest that cannabis use dynamics among adolescents exhibit similar patterns to those observed for alcohol, providing empirical support for extending the TCM to this context. The apparent network effects, documented in prior research and theoretically integral to TCM, are probable mechanisms underlying this collectivity. Adolescent cannabis use is strongly influenced by peer norms and perceptions of harm, which are themselves shaped by broader cultural and policy environments. As cannabis becomes more normalized and its perceived risks decline, these shifts may cascade through social networks, resulting in collective changes in use.

### Study Limitations

Before concluding, it is warranted to point out the major limitations of the study. First, while the school survey data offer a unique long‐term perspective, they are restricted to self‐reports and may be subject to under‐reporting or recall bias. Second, we applied a crude frequency measure of cannabis use. No data on the quantity of consumption were available, and it has been found that the amount consumed makes a difference to cannabis‐related problems—above and beyond the frequency of use [31]. However, constructing a consistent quantity measure across three decades would be challenging. Cannabis comes in many forms (hashish, herbal marijuana, oils, edibles), the relative prevalence of which has shifted over time; further, potency levels have increased substantially [32]. Even if quantity measures had been collected in 1990, their comparability with present‐day data would be doubtful, given the absence of a standardized approach to measuring cannabis quantities in research [31]. Probably for this reason, long‐running surveys such as Monitoring the Future in the USA [33] and the European School Survey Project on Alcohol and Other Drugs (ESPAD) [34] and Young in Norway [35] in Europe also rely on frequency measures. Although an imperfect indicator of cannabis use, frequency seems nevertheless relevant, as demonstrated by its prospective association with a wide range of adverse outcomes [36, 37, 38].

Third, cannabis use was measured as lifetime rather than past‐year prevalence, which may somewhat overestimate recent use. As noted in the Measures section, however, the overlap between lifetime and past‐year use is substantial, and the two indicators track closely over time, making lifetime use a reasonable proxy for recent use. Fourth, the generalizability of the findings may be limited to contexts with similar cannabis policy regimes and youth cultures as Sweden. Future research should explore whether similar patterns of collectivity apply to older age groups and whether they hold in jurisdictions with other legal cannabis regimes. Fifth, this study focused on cannabis users only, in line with the TCM framework. This approach thus highlights variation within the consuming population but does not capture how prevalence and consumption dynamics interact. Our exploratory analyses showed weak and non‐significant correlations between the mean frequency of use among consumers and the proportion of users (both in 9th‐grade students and in 2nd‐year high school students). In contrast, in the case of alcohol, where abstainers are a clear minority, Raninen *et al*. (2022) [39] found a marked negative association in the adult population between the proportion of non‐drinkers and mean consumption among drinkers, suggesting that prevalence and consumption are not independent processes. Similar dynamics could potentially emerge in the context of cannabis if its use were to become more widespread. In any case, the relationship between the proportion of users and mean consumption warrants greater attention in future cannabis research.

## CONCLUSION

The findings support the hypothesis that adolescent cannabis use in Sweden conforms to key predictions of the total consumption model and its extension, the theory of collectivity.

## AUTHOR CONTRIBUTIONS

**Thor Norström**: Conceptualization (equal); data curation (equal); formal analysis (lead); investigation (equal); methodology (lead); project administration (equal); resources (equal); software (equal); supervision (equal); validation (equal); visualization (equal); writing—original draft (lead); writing—review and editing (equal). **Håkan Leifman**: Conceptualization (equal); data curation (equal); funding acquisition (lead); investigation (equal); project administration (equal); resources (equal); software (equal); supervision (equal); validation (equal); visualization (equal); writing—review and editing (equal).

## DECLARATION OF INTERESTS

The authors have no interests to declare.

## Supporting information


**Figure S1.** Lorenz curves with 95% confidence intervals for frequency of cannabis consumption, based on annual data 1990–2023 (excluding 2013) for 9th‐grade students.
**Figure S2.** Lorenz curves with 95% confidence intervals for frequency of cannabis consumption, based on annual data 2004–2023 (excluding 2013 and 2020) for 2nd‐year high school students.

## Data Availability

Data were obtained from a third party and are not publicly available. Information about the procedures to obtain data can be requested from the corresponding author.
