# Supplementary material for: Does the total consumption model apply to cannabis use?
Source: Addiction. 2026 Feb 24;121(7):1721–30. doi: 10.1111/add.70353 (PMC13291080; doi:10.1111/add.70353)
Supplement: Supplementary file 1 — Figure S1. Lorenz curves with 95% confidence intervals for frequency of cannabis consumption, based on annual data 1990–2023 (excluding 2013) for 9th‐grade students. Figure S2. Lorenz curves with 95% confidence intervals for frequency of cannabis consumption, based on annual data 2004–2023 (excluding 2013 and 2020) for 2nd‐year high school students. [file ADD-121-1721-s001.docx]

**Appendix: Figures**


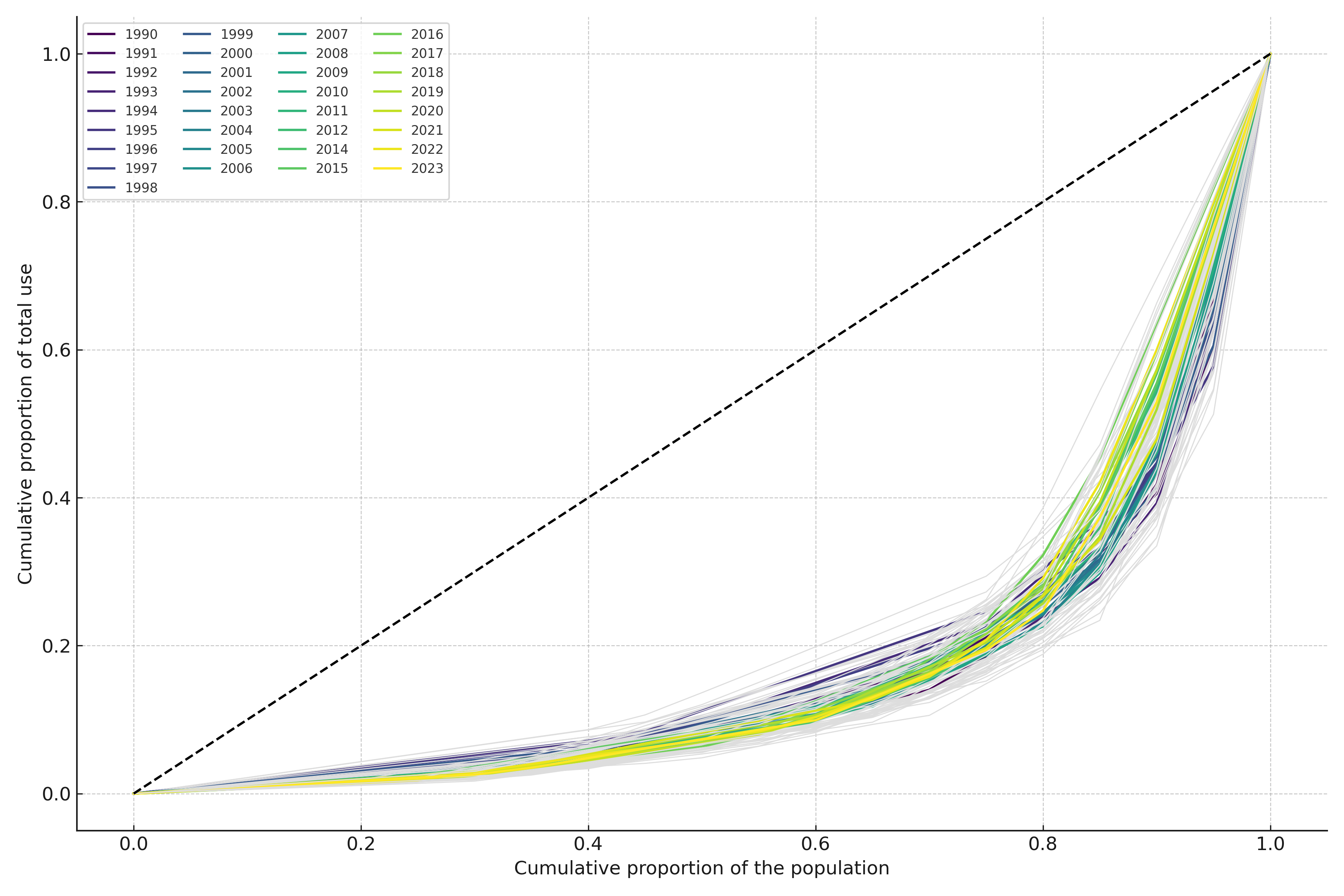


Figure 1. Lorenz curves with 95% confidence intervals for frequency of cannabis consumption. Based on annual data 1990-2023 (excluding 2013) for 9th-grade students.


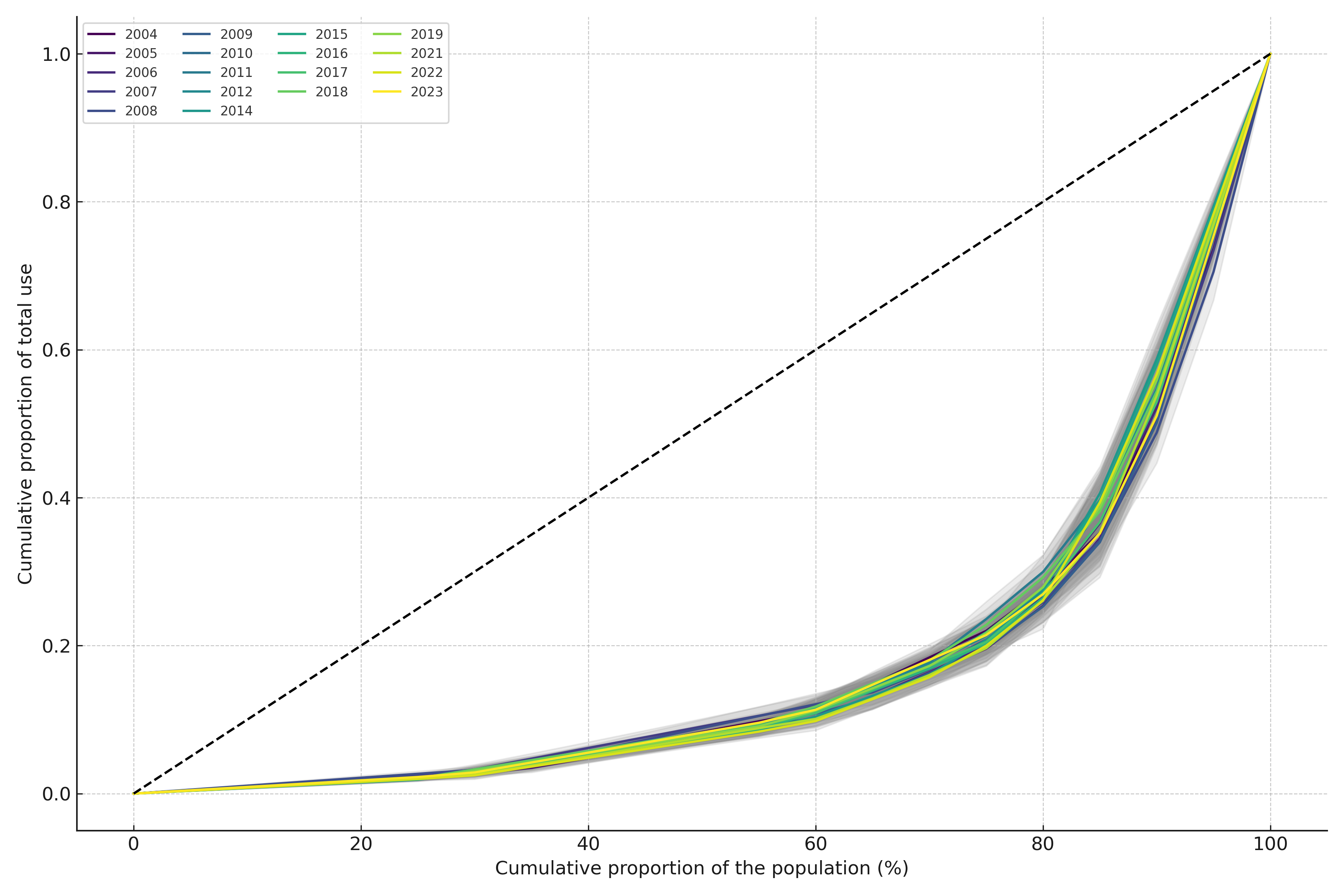


Figure 2. Lorenz curves with 95% confidence intervals for frequency of cannabis consumption. Based on annual data 2004-2023 (excluding 2013 and 2020) for 2nd-year high school students.
